# miR-199a-5p Relieves Obstructive Sleep Apnea Syndrome-Related Hypertension by Targeting HIF-1*α*

**DOI:** 10.1155/2022/7236647

**Published:** 2022-07-27

**Authors:** Chunyan Guo, Minghui Zhang, Wei Su, Min Xu, Shumei Zhao

**Affiliations:** Cardiovascular Center, Beijing Friendship Hospital, Capital Medical University, Beijing, China

## Abstract

**Introduction:**

Obstructive sleep apnea syndrome (OSAS) is related to hypertension. Vascular remodeling is both the pathogenesis and the structural change basis of OSAS-related hypertension. Exploring miRNA functioning in OSAS-related hypertension may offer novel diagnostic and therapeutic targets for controlling hypertension-associated cardiovascular diseases. However, the role of miR-199a-5p in OSAS-related hypertension has not been demonstrated yet.

**Methods:**

In this study, we investigated the role of miR-199a-5p and HIF-1*α* in OSAS-related hypertension by performing *in vitro* cell experiments and *in vivo* animal experiments. Rat aortic smooth muscle cells (A7r5) were cultured under hypoxia as an *in vitro* model. To establish the animal model of OSAS-related hypertension, the rats were under exposure to chronic intermittent hypoxia (CIH) in a hypoxic instrument. The rats were randomly grouped into normal, CIH, CIH+NC, and CIH+miR-199a-5p.

**Results:**

By establishing an animal model, we found decreased miR-199a-5p expression and increased HIF-1*α* expression in OSAS with hypertension. The overexpressed miR-199a-5p could reduce systolic blood pressure and relieve oxidase stress and inflammation. miR-199a-5p treatment could overturn the upregulation of HIF-1*α* and TGF-*β*1 and downregulation of *α*-SMA. Overexpressed miR-199a-5p might attenuate vascular remodeling through HIF-1*α* downregulation. miR-199a-5p/HIF-1*α* may inhibit proliferation of vascular smooth muscle cells under hypoxia.

**Conclusion:**

miR-199a-5p may relieve OSAS-related hypertension by targeting HIF-1*α* and be a novel potential therapeutic target.

## 1. Introduction

In modern society, cardiovascular diseases have become one of the most important causes of mortality and often coexist with sleep disorders, of which obstructive sleep apnea syndrome (OSAS) is the most common type [1]. OSAS is characterized by repeated complete or partial collapse of the upper airway during sleep, causing apnea or hypopnea [2]. OSAS is a systemic disease that can not only cause chronic intermittent hypoxia (CIH) and carbon dioxide retention but also cause or aggravate hypertension, coronary heart disease, arrhythmia, heart failure, diabetes, insulin resistance, and stroke [3, 4]. A number of studies have shown that OSAS is related to hypertension [5, 6]. The prevalence of hypertension in OSAS patients has increased significantly, and the risk of hypertension has increased [6]. OSAS causes or aggravates hypertension through acute physiological effects including increase in the left ventricular afterload, decrease in the left ventricular preload, increase in sympathetic nerve activity, and increase in several neurohumoral factors [7]. Vascular remodeling is both the pathogenesis and the structural change basis of OSAS with hypertension [8].

MicroRNAs (miRNAs) are noncoding endogenous 18–24 nt short RNA molecules and associated with regulation of blood pressure and hypertension [9]. Exploring miRNA functioning in OSAS with hypertension may offer novel diagnostic and therapeutic targets for controlling hypertension-associated cardiovascular diseases. Several miRNAs have been found to be involved in the progress of OSAS, including miR-126a-3p, miR-185, and miR-499 [10–12]. Emerging studies showed that miR-199a-5p plays a regulatory function in heart tissue [13, 14]. Yan et al. found that miR-199a-5p is involved in the progression of heart failure and mediates cardiomyocyte apoptosis [13]. Zhang et al. found that miR-199a-5p is increased in cardiac hypertrophy, and its suppression benefits the cardiomyocytes [14]. However, the role of miR-199a-5p in OSAS with hypertension has not been demonstrated yet.

Dysregulation of hypoxia-inducible factor- (HIF-) 1 activation and HIF-2 suppression caused by CIH has been indicated as a major molecular mechanism for OSAS with hypertension [15]. By previous bioinformatics analysis, we found that HIF-1*α* might be a potential target of miR-199a-5p. Therefore, we focused on the miR-199a-5p/HIF-1*α* signaling pathway. In this study, we investigated the role of miR-199a-5p and HIF-1*α* in OSAS with hypertension by performing *in vitro* cell experiments and *in vivo* animal experiments.

## 2. Methods

### 2.1. Establishment of the Animal Model

The 8-week-old male Sprague-Dawley rats (purchased from Charles River Company, Beijing, China) were kept in specific pathogen-free environment at room temperature. Adequate food and water were provided. The animal experiments were performed with the approval of the animal ethical committee of the Beijing Friendship Hospital. To establish the animal model of OSAS with hypertension, the rats were under exposure to CIH in a hypoxic instrument (JXOC-12; Xinfei Company, Nanjing, China) [12]. The specific procedure was as follows: (a) hypoxia maintenance by adding nitrogen (7%) for 1 min and (b) reoxygenation maintenance by adding oxygen (20%) for 0.5 min. The cycle of a and b was repeated every day from 9 am to 5 pm, and the whole experiments lasted for 56 days. No food or water was given when the rats were under exposure to CIH in the hypoxic instrument. The rats of the control group were kept in the same instrument with normal air environment. The blood pressure (BP) of the caudal artery was measured in triplicate using a blood pressure monitor (ALC-NIBP; Allcote Biotechnology, Shanghai, China) at day 0, day 14, day 28, day 42, and day 56. The animal model was successfully established with the criteria of BP more than 150 mmHg at the end of the experiment.

### 2.2. In Vivo Experiment of miR-199a-5p Treatment

The rats were randomly grouped into (a) normal, (b) CIH, (c) CIH+NC, and (d) CIH+miR-199a-5p (*n* = 6 per group). The recombinant adeno-associated virus (rAAV) empty vector was used as a negative control (NC). Rats in groups c and d were treated with the rAAV empty vector and rAAV-expressing miR-199a-5p (1 × 10^11^ vg/100 *μ*L) through the tail vein before CIH exposure for one time. After 56 days, the rats were sacrificed. The blood, heart tissue, and abdominal aorta were collected.

### 2.3. Quantitative Real-Time PCR (qRT-PCR)

The total RNA extracted from cell and tissue was reversely transcribed into cDNA using commercial kits (Invitrogen, USA). The qRT-PCR was carried out following the manufacturer's instructions. U6 was used for miR-199a-5p, and GAPDH was used for HIF-1*α* as the internal reference.

### 2.4. Western Blot

The antibodies including transforming growth factor-*β*1 (TGF-*β*1; #ab92486), *α*-smooth muscle actin (*α*-SMA; #ab32575), HIF-1*α* (#ab1), and *β*-actin (#ab8226) were purchased from Abcam Company (USA). As previously reported [16], the protein expression of TGF-*β*1, *α*-SMA, HIF-1*α*, and *β*-actin was examined. The quantification was performed using ImageJ software.

### 2.5. Enzyme-Linked Immunosorbent Assay (ELISA)

The collected blood (without anticoagulants to avoid any possible influences) was centrifuged (5000 rpm, 20 min), and the supernatant was collected as serum. The serum was used to measure superoxide dismutase (SOD), malondialdehyde (MDA), high-sensitivity C-reactive protein (hs-CRP), and soluble E-selectin (sE-s) by ELISA kits (purchased from Shanghai Enzyme-linked Biotechnology Company, China). The hs-CRP and s-Es were important inflammatory cytokines [12].

### 2.6. Histopathological Analysis

After fixing in 4% formaldehyde overnight, the section was dehydrated, embedded, and cut into 5 *μ*m thickness. Hematoxylin and eosin (H&E; Sigma-Aldrich Company, USA) staining and Masson's trichrome staining (Maixin Biotechnology, China) were performed to observe the vascular remodeling and fibrosis of the rat heart and abdominal aorta. The Masson-stained positive areas were quantified by ImageJ software for evaluation of fibrosis.

### 2.7. Cell Culture and Transfection

Rat aortic smooth muscle cells (A7r5; Chinese Academy of Sciences, China) were cultured in DMEM (Gibco Company, USA) supplemented with 10% fetal bovine serum (Gibco Company, USA) at 37°C with 5% CO_2_. The miR-199a-5p mimic, miR-199a-5p inhibitor, and corresponding negative controls (NC) were established as follows: miR-199a-5p mimic (forward, 5′-CCG GGA UCC GCA AAC UCA GCU UUA C-3′; reverse, 5′-CGG AAU UCG UGG CGA CCG UGA UAC C-3′), miR-199a-5p inhibitor (3′-CUU GUC CAU CAG ACU UGU GAC CC-5′), mimic NC (forward: 5′-UCA CAA CCU CCU AGA AAG AGU AGA-3′; reverse: 5′-UCU ACU CUU UCU AGG AGG UUG UGA-3′), and inhibitor NC (5′-UCU ACU CUU UCU AGG AGG UUG UGA-3′). Lipofectamine 2000 (Invitrogen, USA) was used to perform cell transfection under hypoxic environment (5% CO_2_, 1% O_2_, and 94% N_2_).

### 2.8. Luciferase Reporter Assay

To generate the HIF-1*α* wide-type (WT) luciferase reporter, the WT HIF-1*α* 3′-UTR which contains the predicted miR-199a-5p binding site was amplified by PCR, followed by insertion into the pmirGLO vector. The mutant HIF-1*α* (Mut) luciferase reporter was generated in the same strategy. Lipofectamine 2000 was used for transfection into A7r5 cells, and the luciferase activity was measured after 48 h.

### 2.9. Cell Proliferation Experiments

BrdU incorporation and colony formation assays were performed. After 24 h of transfection, 10 *μ*M of BrdU was added and incubated for 4 h. After washing for 3 times with PBS, the cells were fixed with 95% ethanol for 20 min. Then, the cells were permeabilized, denatured, and blocked. The cells were incubated with the BrdU antibody (1 : 100; Abcam Company, USA) overnight at 4°C. After secondary antibody incubation and washing, the nuclei were stained with DAPI. The cells were observed using a fluorescence microscope. The BrdU-positive percentage was calculated relative to DAPI-positive cells. The colony formation assay was performed as previously reported [17]. After transfection, the A7r5 cells were cultured for 48 h, followed by fixation and staining by Giemsa (BASO, USA). The cell experiments were repeated for 3 times.

### 2.10. Statistical Analysis

SPSS 22.0 software (IBM Company, USA) was used. The difference between the two groups was analyzed by the *t*-test, and the difference between multiple groups was analyzed by one-way ANOVA. Data were shown as mean ± SD. *P* value < 0.05 was considered statistically significant.

## 3. Results

### 3.1. Decreased miR-199a-5p Expression and Increased HIF-1*α* Expression in the Animal Model of OSAS with Hypertension

**Figure 1:**
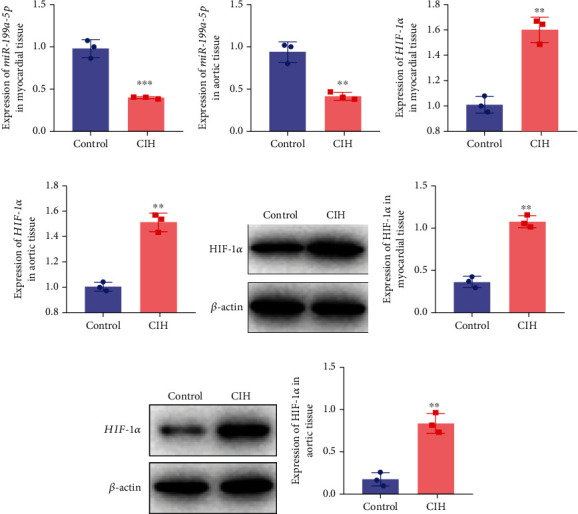
miR-199a-5p and HIF-1*α* expression in the animal model of OSAS with hypertension. mRNA expression of miR-199a-5p in (a) myocardial tissue and (b) aortic tissue by qRT-PCR. mRNA expression of HIF-1*α* in (c) myocardial tissue and (d) aortic tissue by qRT-PCR. Protein expression of HIF-1*α* and quantification in (e, f) myocardial tissue and (g, h) aortic tissue by western blot. CIH: chronic intermittent hypoxia. ^∗∗^*P* < 0.01, ^∗∗∗^*P* < 0.001.

To examine the miR-199a-5p and HIF-1*α* mRNA expression, q-PCR was performed. As shown in Figures 1(a) and 1(b), miR-199a-5p was less expressed in the CIH group in both myocardial and aortic tissues. In contrast, the HIF-1*α* expression in the CIH group was significantly higher than that in the control group (Figures 1(c) and 1(d)). The western blot further confirmed the higher protein expression of HIF-1*α* in the CIH group in myocardial tissue (Figures 1(e) and 1(f)) and aortic tissue (Figures 1(g) and 1(h)).

### 3.2. Overexpressed miR-199a-5p Reduced Blood Pressure

**Figure 2:**
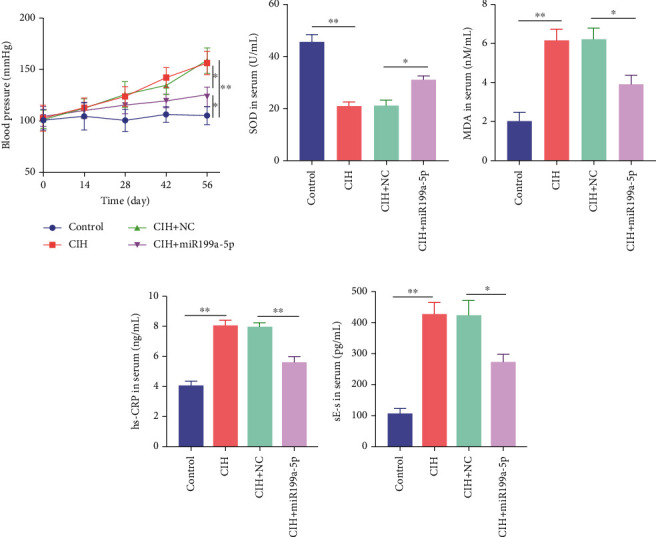
Overexpressed miR-199a-5p reduces blood pressure and relieves oxidase stress and inflammation. (a) Blood pressure in rats with exposure to CIH during 56 days. (b) SOD, (c) MDA, (d) hs-CRP, and (e) sE-s in serum of rats in control, CIH, CIH+NC, and CIH+miR-199a-5p groups. CIH: chronic intermittent hypoxia. ^∗^*P* < 0.05, ^∗∗^*P* < 0.01.

The function of overexpressed miR-199a-5p expression was determined by monitoring the blood pressure in rats with exposure to CIH (Figure 2(a)). The initial systolic blood pressure was normal (100 mmHg). The blood pressure was rising in the three groups including CIH, CIH+NC, and CIH+miR-199a-5p groups after the CIH exposure. At day 56, the blood pressure in the CIH, CIH+NC, and CIH+miR-199a-5p groups was significantly higher than that in the control group. Meanwhile, the blood pressure in the CIH+miR-199a-5p group was lower than that in the CIH and CIH+NC groups (*P* < 0.05), suggesting that the overexpressed miR-199a-5p could reduce systolic blood pressure.

### 3.3. Overexpressed miR-199a-5p Relieved Oxidase Stress and Inflammation

Furthermore, we focused on the central mechanism related to the development of hypertension in OSAS including oxidase stress and inflammation. As shown in Figure 2(b), SOD (an antioxidant enzyme) was reduced in CIH and CIH+NC groups, which was partially rescued by miR-199a-5p treatment. Similarly, MDA (an indicator for oxidative damage) was increased in CIH and CIH+NC groups, which was partially rescued by miR-199a-5p treatment (Figure 2(c)). Two inflammation markers hs-CRP and s-Es were increased in CIH and CIH+NC groups (Figures 2(d) and 2(e)). However, the miR-199a-5p treatment could reduce the increased hs-CRP and s-Es level, induced by CIH exposure. These data indicated that overexpressed miR-199a-5p could relieve oxidase stress and inflammation.

### 3.4. Overexpressed miR-199a-5p Attenuated Vascular Remodeling

**Figure 3:**
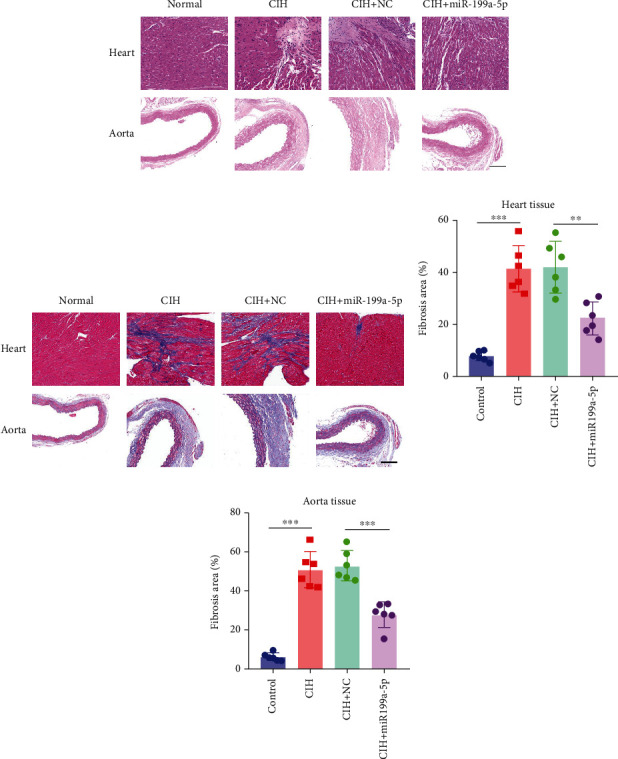
Overexpressed miR-199a-5p attenuated vascular remodeling. (a) Representative H&E staining and (b) Masson staining in the heart and abdominal aorta of rats in control, CIH, CIH+NC, and CIH+miR-199a-5p groups. Scale bar = 25 *μ*m. The fibrosis area of Masson staining in the (c) heart and (d) abdominal aorta. The collagen deposition was stained in blue. CIH: chronic intermittent hypoxia; NC: negative control. ^∗∗^*P* < 0.01, ^∗∗∗^*P* < 0.001. *n* = 6 per group.

To evaluate vascular remodeling, pathological changes and fibrosis in the heart and aorta were observed after H&E and Masson staining. As shown in Figure 3(a), degeneration of cardiomyocytes, myocardial edema, and enlargement of blood vessels in CIH and CIH+NC groups were more obvious than those in the CIH+miR-199a-5p group. Compared with CIH and CIH+NC groups, the CIH+miR-199a-5p group showed more regular arrangement. Besides, miR-199a-5p treatment could decrease the media layer thickness of the aorta. As shown in Figure 3(b), CIH, CIH+NC, and CIH+miR-199a-5p groups exhibited markedly increased collagen deposition. However, the fibrosis area in the CIH+miR-199a-5p group was significantly lower than that in the CIH and CIH+NC groups in both heart tissue (Figure 3(c)) and aorta tissue (Figure 3(d)).

**Figure 4:**
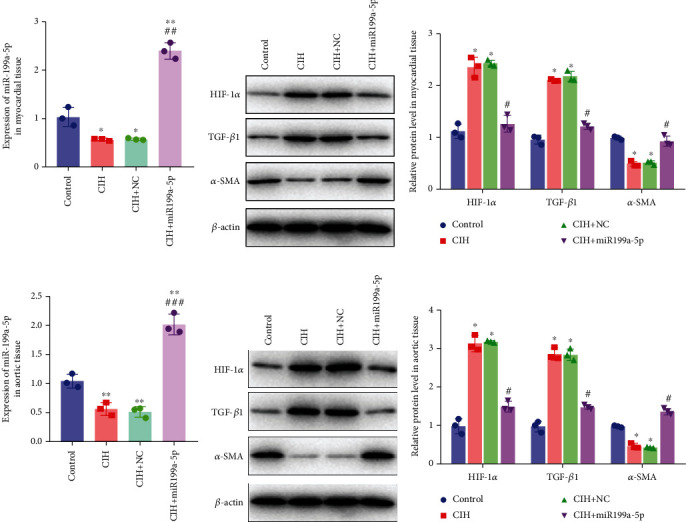
Overexpressed miR-199a-5p attenuated vascular remodeling. (a) mRNA expression of miR-199a-5p in myocardial tissue by qRT-PCR. (b) Protein expression of HIF-1*α*, TGF*β*1, and *α*-SMA in myocardial tissue by western blot and (c) quantification. (d) mRNA expression of miR-199a-5p in aortic tissue by qRT-PCR. (e) Protein expression of HIF-1*α*, TGF*β*1, and *α*-SMA in aortic tissue by western blot and (f) quantification. CIH: chronic intermittent hypoxia; NC: negative control. ^∗^*P* < 0.05, ^∗∗^*P* < 0.01 compared with control; ^#^*P* < 0.05, ^##^*P* < 0.01, and ^###^*P* < 0.001 compared with CIH+NC.

The underlying mechanism was further studied. First, significantly higher miR-199a-5p expression was confirmed in the CIH+miR-199a-5p group in myocardial tissue (Figure 4(a)) and aortic tissue (Figure 4(d)). Compared with the control group, protein expression of HIF-1*α* and TGF-*β*1 (an important cytokine promoting synthesis of the extracellular matrix in vascular remodeling) was significantly higher in the CIH and CIH+NC groups in myocardial tissue (Figures 4(b) and 4(c)) and aortic tissue (Figures 4(e) and 4(f)). At the same time, protein expression of *α*-SMA (a contractile marker in vascular remodeling) was significantly lower in the CIH and CIH+NC groups. Notably, miR-199a-5p treatment could overturn the upregulation of HIF-1*α* and TGF-*β*1 and downregulation of *α*-SMA. Therefore, these results suggested that overexpressed miR-199a-5p might attenuate vascular remodeling through HIF-1*α* downregulation.

### 3.5. miR-199a-5p Targeted HIF-1*α*

**Figure 5:**
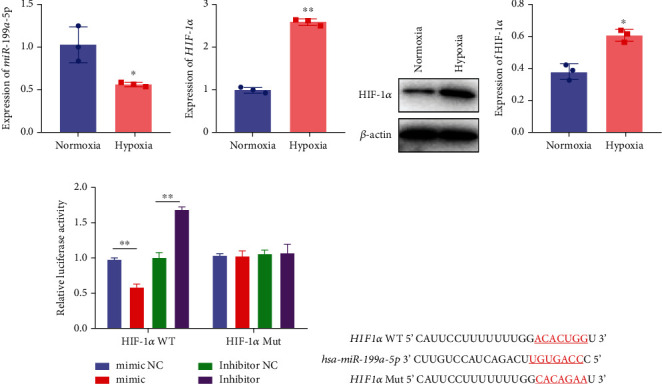
miR-199a-5p targeted HIF-1*α*. mRNA expression of (a) miR-199a-5p and (b) HIF-1*α* in A7r5 cells under normoxia and hypoxia (1% O_2_) by qRT-PCR. (c) Protein expression of HIF-1*α* in A7r5 cells by western blot and (d) quantification. (e) Luciferase reporter assay to validate the putative (f) miR-199a-5p-binding site in the 3′-UTR of HIF-1*α*. WT: wild type; Mut: mutant; NC: negative control. ^∗^*P* < 0.05, ^∗∗^*P* < 0.01.

Based on the results of expression correlation between miR-199a-5p and HIF-1*α*, we further investigate whether miR-199a-5p targets HIF-1*α*. The rat aortic smooth muscle cells under hypoxia exhibited decreased expression of miR-199a-5p (Figure 5(a)) but increased expression of HIF-1*α* (Figures 5(b)–5(d)). Moreover, the luciferase activity in the miR-199a-5p mimic-treated group was significantly reduced in HIF-1*α* wild type (Figures 5(e) and 5(f)). But the luciferase activity in the miR-199a-5p inhibitor-treated group was significantly increased. There were no significant changes in HIF-1*α* mutant. The results indicated that miR-199a-5p targets HIF-1*α* directly.

### 3.6. miR-199a-5p/HIF-1*α* Inhibited Proliferation of Vascular Smooth Muscle Cells under Hypoxia

**Figure 6:**
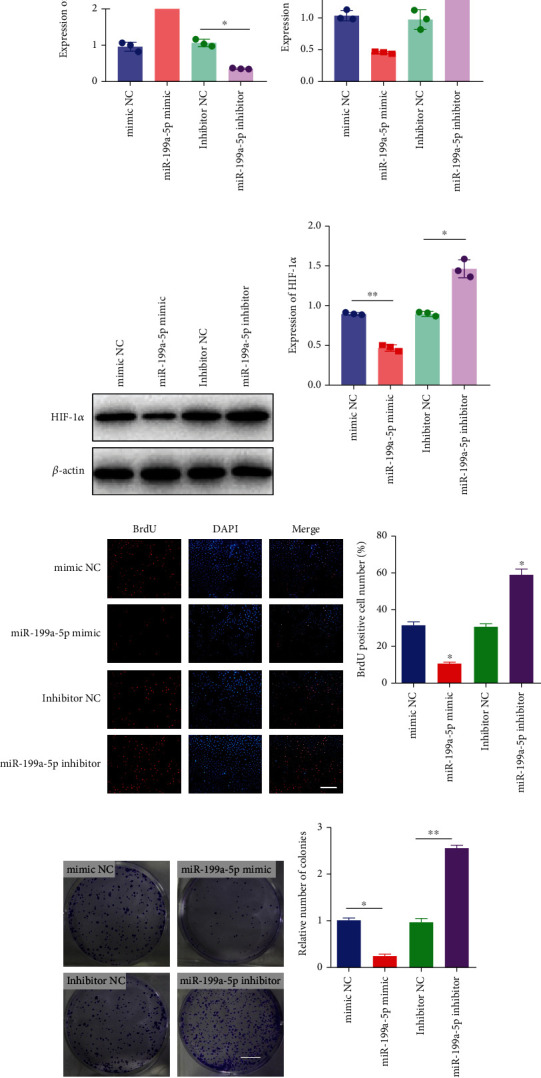
miR-199a-5p/HIF-1*α* inhibited proliferation of vascular smooth muscle cells under hypoxia. mRNA expression of (a) miR-199a-5p and (b) HIF-1*α* in A7r5 cells under normoxia and hypoxia (1% O_2_) by qRT-PCR. (c) Protein expression of HIF-1*α* in A7r5 cells by western blot and (d) quantification. (e) BrdU (red) incorporation in A7r5 cells transfected with the miR-199a-5p mimic, miR-199a-5p inhibitor, and corresponding controls under hypoxia (1% O_2_). Scale bar = 50 *μ*m. (f) Quantification of BrdU-positive cell number. (g) Colony formation assay in A7r5 cells transfected with the miR-199a-5p mimic, miR-199a-5p inhibitor, and corresponding controls under hypoxia (1% O_2_). Scale bar = 50 *μ*m. (h) Quantification of relative number of colonies. NC: negative control. ^∗^*P* < 0.05, ^∗∗^*P* < 0.01, and ^∗∗∗^*P* < 0.001.

miR-199a-5p mimic-treated cells showed significantly higher miR-199a-5p expression while miR-199a-5p inhibitor-treated cells showed significantly lower miR-199a-5p expression (Figure 6(a)). Oppositely, HIF-1*α* expression was reduced in miR-199a-5p mimic-treated cells and increased in miR-199a-5p inhibitor-treated cells (Figures 6(b)–6(d)). Furthermore, the proliferation of vascular smooth muscle cells under hypoxia was examined. As shown in Figures 6(e) and 6(f), the significantly lower BrdU-positive cell number was observed in miR-199a-5p mimic group while the significantly higher BrdU-positive cell number was observed in the miR-199a-5p inhibitor group. The results of colony formation experiment further confirmed this. Thus, miR-199a-5p/HIF-1*α* may inhibit proliferation of vascular smooth muscle cells under hypoxia.

## 4. Discussion

OSAS causes or aggravates hypertension through acute physiological effects. In order to overcome pharyngeal stenosis, the rapid increase in breathing work leads to ineffective breathing effort, which leads to an increase in negative pressure in the thoracic cavity, thereby increasing the left ventricular afterload [3]. The left ventricular diastolic capacity may be weakened, resulting in a further decrease in left ventricular filling. The increase in left ventricular afterload and the decrease in preload also lead to a decrease in stroke volume, which can eventually cause an increase in diastolic blood pressure [4]. At present, OSAS is also considered to be a chronic low-grade inflammatory disease [18]. The inflammatory process leading to vascular endothelial dysfunction plays a pivotal role in the pathogenesis of cardiovascular complications [18, 19]. A number of studies have confirmed that compared with the matched control group, the levels of circulating proinflammatory cytokines, chemokines, and adhesion molecules are increased and significantly decreased after effective treatment, which is likely to be the initiating factor of the inflammatory process [20, 21]. At the same time, cell culture and animal experimental studies have determined that intermittent hypoxia and reoxygenation preferentially activate inflammatory pathways [22]. The induced oxidative stress response may be the main pathophysiological mechanism that causes multiple organ damage [23]. While triggering the oxidative stress response, it also initiates and regulates redox-sensitive signal channels and transcription factors, such as nuclear transcription factors and hypoxia-inducible factors [23]. These transcription factors activate a large number of target genes such as inflammatory factors, which in turn mediates inflammation and cascade effects.

Our results indicated that miR-199a-5p targets HIF-1*α* directly and inhibits proliferation of vascular smooth muscle cells under hypoxia. As known, hypoxia can be activated by a clear mechanism, leading to the expression of many genes encoded proteins, such as erythropoietin, vascular endothelial growth factor and inducible synthase to increase tissue oxygenation [24]. These factors directly increase tissue perfusion and oxygenation and provide adaptability to hypoxia, thereby overcoming the initial damage of hypoxia [24]. HIF-1 is an important transcription factor in the body and an important oxygen-dependent transcriptional activator under hypoxic conditions. HIF-1 is a heterodimer composed of *α* and *β* subunits, of which HIF-1*β* is the basic expression protein, and HIF-1*α* is an oxygen-regulated protein [25]. The protein expression of HIF-1*α* and the activity level of HIF-1 are highly regulated by cellular oxygen concentration.

Under normoxic conditions, specific proline and asparagine residues on HIF-1*α* are hydroxylated to bind to the tumor suppressor gene pVHL, thereby triggering intracellular proteolysis by proteases [26]. HIF-1 is extremely unstable, with a half-life of less than 10 minutes. Under hypoxic conditions, proline hydroxylase activity is inhibited, the tumor suppressor gene pVHL dissociates from HIF-1*α*, the protein degradation pathway is interrupted, and intracellular HIF-1*α* accumulates, activates, and metastasizes in the cytoplasm [27]. After reaching the nucleus, it binds to the HIF-1*β* subunit, recognizes the HIF response element in the promoter of the hypoxia response target gene, and initiates the transcription of the downstream gene [27]. There are more than 40 genes known to be regulated by HIF-1, including erythropoietin, glycolytic enzymes, and VEGF. These genes are involved in various functions such as angiogenesis, erythropoiesis, energy metabolism, apoptosis, and proliferation and play an important role in the physiological responses of cells and organisms [28]. Hypoxia can activate HIF-1 through a well-defined mechanism, resulting in the expression of the proteins encoded by the above genes, thereby increasing tissue perfusion and oxygenation, providing adaptation to hypoxia, thereby overcoming the initial damage of hypoxia [29].

Several miRNAs have been found to be involved in the progress of OSAS. Ding et al. found that chronic OSAS accelerates pulmonary remodeling via TGF-*β*/miR-185/CoLA1 signaling in a canine model [10]. Slouka et al. reported the potential of miR-499 plasmatic level as a biomarker of OSAS [11]. He et al. found that miR-126a-3p targets HIF-1*α* and alleviates OSAS with hypertension [12]. Our study first demonstrated the role of miR-199a-5p in OSAS with hypertension. By establishing an animal model, we found decreased miR-199a-5p expression and increased HIF-1*α* expression in OSAS with hypertension. The overexpressed miR-199a-5p could reduce systolic blood pressure and relieve oxidase stress and inflammation. miR-199a-5p/HIF-1*α* may inhibit proliferation of vascular smooth muscle cells under hypoxia.

## 5. Conclusion

miR-199a-5p may relieve OSAS with hypertension by targeting HIF-1*α* and be a novel potential therapeutic target. The study involving a clinical specimen can further illuminate the role of miR-199a-5p in relieving OSAS with hypertension by targeting HIF-1*α*.

## Data Availability

The datasets used and/or analyzed during the current study are available from the corresponding author upon reasonable request.
